# Knowledge translation for realist reviews: a participatory approach for a review on scaling up complex interventions

**DOI:** 10.1186/s12961-018-0374-1

**Published:** 2018-10-22

**Authors:** Jennifer A. Boyko, Barbara L. Riley, Cameron D. Willis, Lisa Stockton, Dana Zummach, Jon Kerner, Kerry Robinson, Marie Chia

**Affiliations:** 1 Propel Centre for Population Health Impact, University of Waterloo, 200 University Ave West, Waterloo, ON N2L 3G1 Canada; 2 Movember Foundation, P.O. Box 60, East Melbourne, VIC 8002 Australia; 3 Canadian Partnership Against Cancer, 145 King Street West Suite 900, Toronto, ON M5H 1J8 Canada; 4 Public Health Agency of Canada, 130 Colonnade Road A.L. 6501H, Ottawa, ON K1A 0K9 Canada; 5 Faculty of Health, Arts and Design, Swinburne University of Technology, John Street, Hawthorn, VIC 3122 Australia

**Keywords:** realist reviews, knowledge translation, scaling up, complex interventions, chronic disease prevention, system planning, public health, population health, evidence-informed decision-making

## Abstract

**Background:**

Knowledge syntheses that use a realist methodology are gaining popularity. Yet, there are few reports in the literature that describe how results are summarised, shared and used. This paper aims to inform knowledge translation (KT) for realist reviews by describing the process of developing a KT strategy for a review on pathways for scaling up complex public health interventions.

**Methods:**

The participatory approach used for the realist review was also used to develop the KT strategy. The approach included three main steps, namely (1) an international meeting focused on interpreting preliminary findings from the realist review and seeking input on KT activities; (2) a targeted literature review on KT for realist reviews; and (3) consultations with primary knowledge users of the review.

**Results:**

The international meeting identified a general preference among knowledge users for findings from the review that are action oriented. A need was also identified for understanding how to tailor findings for specific knowledge user groups in relation to their needs. The literature review identified four papers that included brief descriptions of planned or actual KT activities for specific research studies; however, information was minimal on what KT activities or products work for whom, under what conditions and why. The consultations revealed that KT for realist reviews should consider the following: (1) activities closely aligned with the preferences of specific knowledge user groups; (2) key findings that are sensitive to factors within the knowledge user’s context; and (3) actionable statements that can advance KT goals, activities or products. The KT strategy derived from the three activities includes a planning framework and tailored KT activities that address preferences of knowledge users for findings that are action oriented and context relevant.

**Conclusions:**

This paper provides an example of a KT strategy for realist reviews that blends theoretical and practical insights. Evaluation of the strategy’s implementation will provide useful insights on its effectiveness and potential for broader application.

**Electronic supplementary material:**

The online version of this article (10.1186/s12961-018-0374-1) contains supplementary material, which is available to authorized users.

## Background

Knowledge created through application of the scientific method requires effort to translate into action. This process, which involves identifying who (e.g. healthcare providers, public health professionals, government decision-makers) potential knowledge users might be and how they might be involved in the process, is referred to as knowledge translation (KT). A commonly cited definition of KT is “*a dynamic and iterative process that includes synthesis, dissemination, exchange and ethically-sound application of knowledge to improve health, provide more effective health services and products, and strengthen the health care system*” [1]. The science of KT has developed significantly over the past two decades in response to growing demand for ensuring research findings are used to inform decision-making in clinical, organisational and policy contexts [2]. Theoretical frameworks exist that help to explain the knowledge-to-action process [3, 4] and applied research has been performed to understand the most effective ways to achieve desired KT outcomes in public health [5], healthcare [6, 7] and public policy [8].

A key part of KT that has been studied is knowledge synthesis or “*the contextualization and integration of research findings of individual research studies within the larger body of knowledge on the topic*” [1]. Although most syntheses will proceed through similar stages (e.g. form review team, formulate question, search and screen evidence, etc.), specific methods and data will be used depending on the research questions and intended use [9, 10]. For example, aggregative reviews may collect and combine quantitative data from primary studies in order to test hypotheses and make cause and effect statements about intervention effectiveness (i.e. what is the effect of X on Y?) [10]. Configurative reviews may collect, organise and interpret different types of data (qualitative, quantitative) in order to answer questions that help understand experiences and meanings, and generate theory about the world (i.e. what do we know about X and Y?) [10]. Realist reviews may aggregate and configure data to offer explanations about how an intervention works in particular contexts (i.e. what works for whom, in what circumstances, in what respects and how?) [10, 11]. Realist reviews, in particular, can be used by decision-makers to gain deep understanding about complex social interventions that can be used when planning and implementing national, regional or local level programmes [11]. To do so, realist approaches focus on understanding the contexts (i.e. circumstances that surround the implementation of a given intervention or phenomena), mechanisms (i.e. underlying processes that are sensitive to context and influence outcomes) and outcomes (i.e. effects that occur over time) [11–14]. Context, mechanism and outcome (CMO) configurations explain how specific actions interact with context, which in turn activate specific mechanisms and lead to specific outcomes.

The availability of findings from realist reviews, or any type of knowledge synthesis, does not assure that the intended benefits will be achieved or that knowledge-users will use the findings [15, 16]. Researchers employing co-creation approaches (e.g. integrated knowledge translation, community-based participatory research) report that research is more likely to be used in health systems by knowledge users [17]. However, the empirical knowledge base does not offer any clear guidance about the extent to which use extends beyond those involved in co-production processes, how knowledge users are actively engaged in dissemination planning, or the extent to which engagement actually leads to use. Strategies are required to address barriers to research use, such as access, timeliness, user-friendliness and relevance of evidence [8, 18], faced by decision-makers. In order to overcome such barriers, researchers have examined ways to effectively package the results from systematic reviews of intervention effectiveness to meet the needs of patients [19–21], healthcare professionals [22–24] and policy-makers [25]. For example, decision support aids and plain language summaries have been found to support the uptake of systematic review findings by patients and providers, respectively [21]. Similarly, we know that health system managers and policy-makers prefer to receive the findings of systematic reviews in the form of evidence briefs that present key messages and findings up front [26].

In general, there is some empirical evidence to guide decisions about the KT strategy for systematic reviews of effectiveness targeting healthcare professionals, policy-makers and senior managers [27]. This evidence includes understanding about who the knowledge should be transferred to, by whom, how, and with what effect [27]. This understanding can inform the development of any KT strategy. However, the literature is silent on whether or how to tailor such guidance for different types of reviews and on the influence of tailoring on use. Reviews differ from one another in terms of purpose, methods and nature of findings. Realist reviews are unique in that the aim is to develop explanatory insight (i.e. theory) to explain how an intervention works in practice, for whom and in what contexts [10, 11]. The broad scope of realist reviews will often require describing, analysing and synthesising a diverse and expansive range of document types (e.g. peer-reviewed research, administrative data, annual reports, legislative documents, transcripts) [28]. Although quality standards for reporting realist review findings are available [14], these standards do not explain how to translate theory-based findings for knowledge users. This paper aims to inform the development of KT for realist reviews, especially in supporting the intended influence on policy and practice. Members of the authorship team (CW, BR, LS) completed a realist review on pathways for scaling up complex public health interventions [29], and expanded the study by developing a KT strategy for the review. Discussion about KT took place early on in the review process. However, as findings started to emerge, it became clear that (1) the participatory approach was not sufficient to support use, and (2) existing KT frameworks did not provide sufficient guidance for tailoring the results. A strategy was needed to translate the findings of the realist review for use by different knowledge user groups. The focus of this paper is on the KT strategy – both the development process and the products.

### Realist review on scaling up

Despite much success, many public health interventions fail to reach those most in need. This is particularly so for complex public health interventions, including in chronic disease prevention, that involve multi-component and multi-level efforts tailored to the contexts in which they are delivered [30]. A realist review was undertaken to understand how and under what conditions complex public health interventions may be scaled up to benefit more people and populations [29]. Scaling up refers to the process of improving coverage of and equitable access to innovations with demonstrated effectiveness on a smaller scale [30, 31]. The realist review focused on three cases of successfully scaled up interventions in order to understand pathways for scaling up. A systematic search for published and grey literature related to each case was carried out. Relevant literature informed the creation of CMO configurations, which were then compared, contrasted and interpreted alongside the review’s programme theory and diffusion of innovation theory. The synthesis process was highly participatory, engaging knowledge users and those with content and methods expertise. Engagement strategies with experts and knowledge users involved ongoing email communications and periodic teleconferences over the course of the study, and an in-person workshop.

**Fig. 1:**
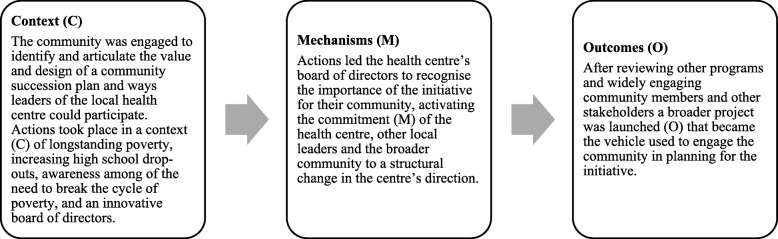
Context, mechanism and outcome (CMO) configuration to illustrate how commitment may be activated to renew and regenerate a complex community initiative

The review found that four core mechanisms are commonly activated when scaling up complex public health interventions, namely awareness, commitment, confidence and trust. The mechanisms were activated by actions to renew and regenerate interventions, and documenting success. Specific actions included building partnerships, conducting evaluations, engaging political support and adapting funding models, all of which interact with contextual conditions. Resulting scaling up outcomes were the engagement of new communities, new or amended legislation, or adding new funding partners. Figure 1 is an example of one CMO configuration that also highlights the complexity of realist review findings. The example illustrates how the mechanism of commitment was activated by efforts to design a community succession plan within a context of long-standing poverty and high rates of high-school non-completion, eventually leading to a project focused on renewing and regenerating the community. The realist review advances understanding of the practice and theory of scaling up complex interventions by demonstrating that practitioners may benefit from a number of coordinated efforts, including conducting or commissioning evaluations at strategic moments, mobilising local and political support through relevant partnerships, and promoting ongoing knowledge exchange in peer-learning networks.

## Methods

### Description of KT strategy development

**Fig. 2:**
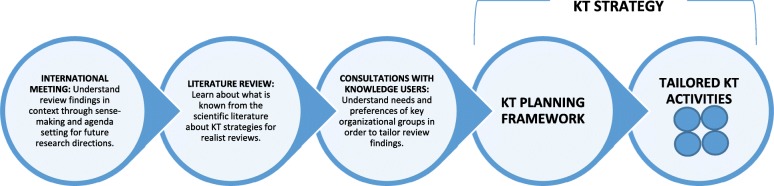
Approach used to develop the knowledge translation (KT) strategy

Figure 2 illustrates the approach to KT strategy development. It was highly participatory, with members of the realist review’s knowledge user panel working closely with the research team to complete the three main activities, and translate them into the two components of the KT strategy. The approach was also informed by a subset of KT frameworks with relevance to public health [32–38]. Common elements among these frameworks (e.g. the importance of considering context given that specific factors can support or work against knowledge to action processes in different settings or circumstances) are reflected in the activities and components described below.

#### International meeting

The initial step was an international meeting held in Toronto, Canada, that focused on scaling up cancer and chronic disease prevention interventions for population health impact. The idea for the in-person meeting came from knowledge users participating in the realist review who were interested in learning from each other about scaling up through practical examples. The objectives were to (1) compare and contrast preliminary findings from the review with knowledge user experiences; (2) refine KT plans for study findings (i.e. the focus of this paper); and (3) identify additional scaling up initiatives that may be promising to study as natural experiments.

A strategic mix of Canadian and international research, policy and practice leaders, with diverse experiences in scaling up cancer and chronic disease interventions were invited to participate in the meeting. Twenty-four individuals participated, including knowledge users with interest in the realist review results (*n* = 10), academics with expertise in scaling up and/or realist review methods (*n* = 8), and review team members (*n* = 6). Four international participants attended via videoconference. The 1-day meeting agenda (Additional file 1) included a panel discussion, presentations, small group work and facilitated large group discussions. A more detailed description of the international meeting is available in the full meeting report [39].

#### Literature review

A targeted review of the literature was carried out following the meeting. The goal was to identify at least a subset of papers that focused specifically on understanding how to optimise the impact of realist reviews. Keyword searches were conducted in Scopus, Web of Science and PubMed. Searches combined terms related to KT (“knowledge utilization/uptake/exchange/implementation”, “use of research findings”, “research utilization”) and realist reviews (“realist synthesis/evaluation”, “rapid realist review”). Members of the research team also nominated papers. Relevant papers were considered those that identified and described specific strategies (products, activities, approaches) used (or proposed) to translate realist review findings into action. One team member screened titles and abstracts as searches were completed. A different team member retrieved the full text for 33 articles and assessed them for relevance. Four articles were deemed relevant and, from these, data were extracted on details related to KT strategy.

#### Consultations with knowledge users

Discussion from the international meeting and findings from the literature review informed consultations with knowledge users to learn more about their KT needs and preferences. The interviews did not explore views about the KT strategy for realist reviews more generally. Three individuals that attended the international meeting and that represented key knowledge user organisations were invited and participated. The consultations were semi-structured and took place on the telephone. Topics explored included (1) primary audiences for the realist review within their organisation and findings most pertinent to them; (2) key factors (e.g. individual characteristics, organisational conditions) that might influence use of the review results; (3) preferences for KT activities; and (4) use of realist methodology to explore patterns and pathways for scaling up population interventions. The full set of questions is available in Additional file 1. One member of the research team conducted the consultations and made extensive notes during each call. The consultations were not audio-recorded or transcribed. Instead, a summary of each consultation was completed and sent to each consultee to confirm that their ideas were captured accurately.

## Results

Contributions to the KT strategy from each activity in the planning process are summarised below. The KT strategy, which includes a planning framework and KT activities tailored to meet the needs of specific knowledge user groups, is also described.

### International meeting

The meeting discussion pertaining to KT plans for the realist review on scaling up reflected four main areas. First, different knowledge user groups that were represented at the meeting (e.g. government and non-government organisations) were interested in different findings from the review. For example, one organisation was particularly interested in discussion about how to make scale up happen given their mandate to fund projects that might be scaled up. Another organisation expressed interest in learning about the role of networks within communities engaged in scale up initiatives. Second, knowledge users expressed interest in learning about the findings of the review through a KT strategy tailored to their needs and preferences. Several potential target audiences for the review’s results were identified (beyond the knowledge users in attendance at the meeting), including specific characteristics of each audience helpful in tailoring KT to meet their needs. These characteristics include preferences for specific KT activities, appropriate engagement strategies and timing of dissemination efforts, as well as value placed on establishing personal relationships with intended knowledge users. Third, there was acknowledgement among meeting participants (researchers and knowledge users) that KT for realist reviews is not well understood, and could be advanced by sharing the experience of developing a KT strategy for the realist review with others. Fourth, actions arising from the meeting included seeking further understanding about KT strategies for realist reviews from published literature as well as the KT needs and preferences of potential knowledge users.

### Review of the literature

Of the four papers reviewed in-depth, two were realist reviews, one was a protocol for a realist review, and another focussed on describing rapid realist reviews (i.e. a realist approach to knowledge synthesis that emphasises producing a time-sensitive product responsive to policy-makers’ information needs). Specific types of KT activities identified in the papers included healthcare planning frameworks and tools to support decision-makers [40, 41], publications for academic and lay audiences [41], conference presentations [41], tailored workshops and presentations for knowledge users (e.g. policy-makers) [41, 42], and knowledge brokering to understand policy and academic perspectives and develop recommendations that are sensitive to local contexts [43]. Consistent with realist methodology, each paper described knowledge user engagement throughout the review process. The included papers articulated the aim of the KT strategy in different ways. Two papers indicated that their KT efforts would help them achieve goals related to the purpose of the research [41, 43]. One paper was more specific in how their findings would be used, stating that findings would inform the development of a new intervention [40]. The methodological paper identified several practical considerations for enabling KT for realist reviews such as engaging knowledge users with demanding time constraints (e.g. agency or government staff) in a reference group rather than an active member of the synthesis team [43]. Data extraction from the four papers is available in Additional file 1.

### Consultations with primary knowledge users

The input received from knowledge users provided specific guidance for developing tailored KT activities for the scaling up review and preferences for KT associated with realist reviews in general. A focus on learning about key findings related to context was a common theme across the consultations. This includes identifying challenges (e.g. poverty, labour disruptions) that different groups may face when implementing programmes and recognising that these challenges may overshadow mechanisms. One knowledge user specifically noted the importance of identifying the preferences of senior managers in light of contextual factors that may impede the usability of the results. This could include preferences for when to disseminate results to avoid peak organisational times (e.g. fiscal year end) or what key messages to relay in order to emphasise how the results could further action and policy.

Another common theme across the consultations was the preference for findings that are action oriented. The knowledge users expressed interest in receiving review findings in the form of actions they can take to advance specific goals in a scaling up process. They expressed keen interest in the actions outlined in the realist review and not the mechanisms that relate to underlying processes. Actions were considered practical and could be used to inform their investments and planning. Examples of actions from the review included commissioning external evaluations, adapting a funding model to engage community-based organisations, and developing mergers between existing organisations with shared objectives.

Input received from knowledge users about tailoring KT activities included preference for evidence briefs as a KT product, particularly in contexts with high staff turnover and where practical guidance was valued. Other preferences included facilitated group discussions or presentations that describe study findings and provide opportunity for programme staff and managers to ask questions. Combining a presentation with an evidence brief was suggested as being particularly useful.

Knowledge users also explained that the realist methodology and terminology may not be well understood by practitioners and should be avoided in dissemination efforts. A suggestion to address this was providing a rubric with key terms along with any results.

#### KT strategy

Results from the three activities described above were integrated into a KT strategy for the realist review on scaling up. The KT strategy has two components, namely a planning framework and a set of KT activities tailored to knowledge user needs. The KT planning framework is presented in Box 1; it includes a set of KT principles and components intended to support the development of tailored KT activities that meet the needs of specific knowledge user groups.

Initial priority for using the framework was with three knowledge users who were most actively engaged in the realist review and the KT planning process. Table 1 represents an illustrative example of how this was done for one knowledge user group by using the input received during their consultation. The example highlights how tailored KT activities address the needs of three particular audiences with whom the knowledge user interacts. The planning framework helped the knowledge users identify the potential target audiences for the review’s findings, as well as key messages and KT products most appropriate for each.

**Table 1:** Use of the knowledge translation (KT) planning framework to develop tailored products or activities

TARGET AUDIENCE (who, as well as key characteristics and contextual considerations)	KEY MESSAGES (source and focus of messages)	KT ACTIVITIES (products, processes, events, strategic communications, etc.)	OUTCOMES (desired results)
Programme staff/teams within knowledge user organisations	Concrete examples of how to support activation of the four mechanisms (i.e. findings of facilitators/barriers) Relate examples to organisational experience (i.e. how to scale-up knowledge gained from past complex initiatives) Practical implications of findings (e.g. how findings can support evaluation of scale-up activities) Findings related to complex elements of the three case studies (General: avoid jargon, clarify main concepts, align with current language, avoid directive language)	Facilitated group discussions that encourage learning among participants Short visually appealing materials (e.g. infographics, evidence brief) Stories that illustrate key findings Relevance of the results for the health sector	Use of findings in funding decisions Improved communication with grant applicants Strengthened grant applications
Senior management staff within knowledge user organisations	Focus on policy relevance not implementation Acknowledge that scaling up complex initiatives is challenging, but that working with many partners in different sectors is necessary to impact policy and deliver interventions to individuals in communities Use examples that demonstrate impact and why findings are important in order to raise their significance amongst competing issues Situate findings among other current studies that may be known to staff	Identify and use champion to deliver messages Have ‘ready to go’ information about results that can be adapted in order to respond to requests for information in relation to emerging issues Short written materials (e.g. policy brief or high-level synthesis of the findings) Short visually appealing materials (e.g. infographics)	Requests for additional information about scaling up Findings used to provide justification for programmatic or policy decisions
Policy-makers external to knowledge user organisations	Understanding of what scaling up means and how it gets operationalised, what the challenges are (General: avoid jargon, clear take-home messages)	Short written materials (e.g. policy brief or high-level synthesis of the findings)	Requests for additional information about scaling up Findings used to provide justification for programmatic or policy decisions

## Box 1 Knowledge translation (KT) planning framework

Foundational principles:

- Interaction and feedback among knowledge producers and users, as well as among different types of KT activities (passive, purposeful, action oriented) supports integration across individual, organisational and system contexts.
- KT planning engages target audiences as partners in developing and implementing KT strategy to ensure plans meet needs and interests, and include KT activities that encourage the use of research results.
- Target audiences are understood by considering specific characteristics related to knowledge use, as well as broader contextual considerations that influence use.
- Key messages consider aspects of the message itself, as well as appropriate target audiences, messengers, activities and anticipated effects.
- Using a variety of KT activities supports the uptake of research findings across the public health system.

Main components:

- Target audiences – Broad categories of people, as well as specific individuals, organisations and networks within these, to whom KT activities are targeted.
- Key messages – Specific pieces of knowledge, as well as their sources, arising from research that are meaningful and relevant to target audiences.
- KT activities – Approaches and actions (e.g. products, processes, events and strategic communications) that communicate key messages to target audiences, and facilitate their interpretation and use.
- Outcomes – Anticipated effects of KT strategy and activities relative to specific audiences. Used to devise an evaluation plan that details specific evaluation questions, measures and methods.

## Discussion

We described development of a KT strategy for a realist review on scaling up complex public health interventions. Our approach to developing the strategy, as well as the strategy itself, was an extension of the original study and emerged as the study team learned about the knowledge needs and learning preferences of different knowledge user groups. The process included three main activities, namely an international meeting, a literature review and consultations with knowledge users. Insight gained from each of these activities culminated into a KT strategy consisting of a planning framework to support development of tailored KT activities that meet the needs of different knowledge user groups. The experience we document and share through this paper represents a practical contribution to the field of KT. Most notably, our experience highlights the importance of making KT explicit and the value of participatory approaches in KT planning. This paper also provides guidance about how to tailor findings from realist reviews to specific knowledge users.

Not necessarily unique to realist reviews, our experience demonstrates the importance of making KT planning tasks explicit from the outset, while maintaining openness to adapting plans [44]. Early discussions about KT helped elicit rich perspectives from both researchers and knowledge users about the KT strategy. As the review progressed and findings emerged, the KT strategy became formalised. It helped to have distinct steps that drew in practical experience (e.g. international meeting, consultations with knowledge users), and information from the KT literature. Our experience is consistent with other literature that found integrating and translating the insights from specific planning steps helped produce practical output [40–43] such as the KT planning framework and its application in the table of tailored KT products and activities.

The value of participatory approaches in KT planning is also apparent from our experience. This insight emerged through a series of interactions with knowledge users, throughout the completion of the realist review itself, and the KT planning process. The ability of knowledge users to identify such diverse and nuanced needs seemed to grow as engagement progressed. It may be that synergy accumulated among the research team through on-going successful collaboration, thus increasing the quality of outputs and outcomes of engagement over time [17]. While the use and benefits of participatory processes in health research are well-known [45, 46], the added value of engagement in specific stages of completing a realist review, including KT strategy development, are not specifically addressed in the research literature. Research related to the qualities of collaborative working (e.g. reciprocity, equality of partners) in co-produced research studies that support knowledge generation and translation may be a helpful starting point [47, 48].

More specific to realist reviews, we learned that findings need extensive tailoring, not only for different organisations, but also for different audiences within each organisation. For example, one organisation’s preferences for tailoring results included ensuring key messages ‘stick’ within the organisation amidst high staff turnover by embedding key messages in new staff training. Another organisation preferred that KT messages be tailored by keeping in mind a recent organisational shift from knowledge generation to better utilisation of existing evidence. Programme staff from knowledge user organisations would benefit from findings to support them in implementing or evaluating efforts to scale up knowledge gained from past complex initiatives, whereas senior managers would benefit more from learning about policy implications. The detailed and diverse needs expressed by our knowledge users may also be a result of the nature of findings from realist reviews. The complexity of realist review findings prompted considerable thought about what was needed, by whom, when and why.

Also specific to realist reviews was a very strong preference for action-oriented findings. We learned from the meeting and interviews that knowledge users are not interested in the theory building that is central to realist reviews. It became clear that a KT strategy should focus on findings that guide actions of knowledge users, including the contextual conditions under which actions are likely to be most successful [42, 43]. The specific mechanisms triggered by actions and changes in context were not considered as useful to the knowledge users involved in the realist review. Technical or realist jargon must also be avoided in messages. These findings reinforce what is known about effective KT [49, 50], extending insights to realist reviews.

### Limitations

This paper draws on the experience of one research team in integrating different forms of knowledge to create a KT strategy for a realist review. While it is well known that knowledge user differences need to be borne in mind when planning KT, this paper provides a practical example of how this can be done. The approach was applied to a realist review on scaling up complex interventions; using the approach with other topics would help to discern its transferability. Implementing and evaluating the influence of the KT activities is also needed to understand the effectiveness of the approach to KT planning.

## Conclusion

This paper begins to fill a gap in knowledge about KT for realist reviews and provides practical guidance for those engaged in scaling up complex interventions in public health. Future research might build on this work and explore the usefulness of realist reviews, including preferences of specific knowledge user groups (e.g. government policy-makers) for receiving the findings. Additional application and adaptation of our approach with other topic areas, by different teams, and in different contexts will help to discern its utility. An important next step is implementation and evaluation of the KT strategy.

## Additional file

Additional file 1: International meeting agenda. Questions from consultations with knowledge users. Summary of papers considered relevant to understanding the knowledge translation (KT) strategy for realist reviews. (DOCX 30 kb)

## Abbreviation

CMO: Context, mechanism and outcome
KT: Knowledge translation

## References

[1] Canadian Institute for Health Research. About Knowledge Translation. http://www.cihr-irsc.gc.ca/e/29418.html. Accessed 13 Sept 2018.

[2] Almeida C, Báscolo E (2006). Use of research results in policy decision-making, formulation, and implementation: a review of the literature. Cad Saude Publica.

[3] Davison CM, Ndumbe-Eyoh S, Clement C (2015). Critical examination of knowledge to action models and implications for promoting health equity. Int J Equity Health.

[4] Tabak RG, Khoong EC, Chambers D, Brownson RC (2012). Bridging research and practice: models for dissemination and implementation research. Am J Prev Med.

[5] LaRocca R, Yost J, Dobbins M, Ciliska D, Butt M (2012). The effectiveness of knowledge translation strategies used in public health: a systematic review. BMC Public Health.

[6] Gagliardi AR, Berta W, Kothari A, Boyko J, Urquhart R (2016). Integrated knowledge translation (IKT) in health care: a scoping review. Implement Sci.

[7] Scott SD, Albrecht L, O’Leary K, Ball GD, Hartling L, Hofmeyer A (2012). Systematic review of knowledge translation strategies in the allied health professions. Implement Sci.

[8] Oliver K, Innvar S, Lorenc T, Woodman J, Thomas J (2014). A systematic review of barriers to and facilitators of the use of evidence by policymakers. BMC Health Serv Res.

[9] Tricco AC, Tetzlaff J, Moher D (2011). The art and science of knowledge synthesis. J Clin Epidemiol.

[10] Gough D, Thomas J, Oliver S (2012). Clarifying differences between review designs and methods. Syst Rev..

[11] Pawson R, Greenhalgh T, Harvey G, Walshe K (2005). Realist review--a new method of systematic review designed for complex policy interventions. J Health Serv Res.

[12] Astbury B, Leeuw FL (2010). Unpacking black boxes: mechanisms and theory building in evaluation. Am J Eval.

[13] Hedstrom P, Ylikoski P (2010). Causal mechanisms in the social sciences. Annu Rev Sociol.

[14] Wong Geoff, Greenhalgh Trish, Westhorp Gill, Pawson Ray (2014). Development of methodological guidance, publication standards and training materials for realist and meta-narrative reviews: the RAMESES (Realist And Meta-narrative Evidence Syntheses – Evolving Standards) project. Health Services and Delivery Research.

[15] Centre for Reviews and Dissemination (2008). Systematic Reviews: CRDs Guidance for Undertaking Reviews in Health Care.

[16] Chambers D, Wilson PM, Thompson CA, Hanbury A, Farley K, Light K (2011). Maximizing the impact of systematic reviews in health care decision making: a systematic scoping review of knowledge-translation resources. Milbank Q..

[17] Jagosh J, Macaulay AC, Pluye P, Salsberg J, Bush PL, Henderson J (2012). Uncovering the benefits of participatory research: implications of a realist review for health research and practice. Milbank Q..

[18] Wallace John, Nwosu Bosah, Clarke Mike (2012). Barriers to the uptake of evidence from systematic reviews and meta-analyses: a systematic review of decision makers’ perceptions. BMJ Open.

[19] Brehaut JC, Eva KW (2012). Building theories of knowledge translation interventions: Use the entire menu of constructs. Implement Sci.

[20] Santesso N, Rader T, Nilsen ES, Glenton C, Rosenbaum S, Ciapponi A (2015). A summary to communicate evidence from systematic reviews to the public improved understanding and accessibility of information: a randomized controlled trial. J Clin Epidemiol.

[21] Rader T, Pardo Pardo J, Stacey D, Ghogomu E, Maxwell LJ, Welch VA (2014). Update of strategies to translate evidence from cochrane musculoskeletal group systematic reviews for use by various audiences. J Rheumatol.

[22] Perrier L, Persaud N, Thorpe KE, Straus SE (2015). Using a systematic review in clinical decision-making: a pilot parallel, randomized controlled trial. Implement Sci.

[23] Rosenbaum SE, Glenton C, Nylund HK, Oxman AD (2010). User testing and stakeholder feedback contributed to the development of understandable and useful summary of findings tables for Cochrane reviews. J Clin Epidemiol.

[24] Maguire LK, Clarke M (2014). How much do you need: a randomised experiment of whether readers can understand the key messages from summaries of Cochrane Reviews without reading the full review. J R Soc Med.

[25] Petkovic J, Welch V, Tugwell P (2015). Do evidence summaries increase policy-makers’ use of evidence from systematic reviews: a systematic review protocol. Syst Rev.

[26] Ellen ME, Lavis JN, Wilson Michael G, Grimshaw J, Haynes RB, Ouimet M, Raina P (2014). Health system decision makers’ feedback on summaries and tools supporting the use of systematic reviews: a qualitative study. Evid Policy A J Res Debate Pract.

[27] Grimshaw JM, Eccles MP, Lavis JN, Hill SJ, Squires JE (2012). Knowledge translation of research findings. Implement Sci.

[28] Berg R, Nanavati J (2016). Realist review: current practice and future prospects. J Res Pract.

[29] Willis CD, Riley BL, Stockton L, Abramowicz A, Zummach D, Wong G (2016). Scaling up complex interventions: insights from a realist synthesis. Health Res Policy Syst.

[30] Edwards N. Scaling up health innovations and interventions in public health: a brief review of the current state of the science. Washington, DC: Conference to Advance the State of the Science and Practice on Scale-up and Spread of Effective Health Programs; 2010. http://www.ihi.org/education/Documents/ProgramMaterials/ScaleUpBlog/7a_Commissioned_Paper2_Public_Health.doc. Accessed 13 Sept 2018.

[31] Mangham LJ, Hanson K (2010). Scaling up in international health: what are the key issues?. Health Policy Plan.

[32] Butler H, Bowes G, Drew S, Glover S, Godfrey C, Patton G (2010). Harnessing complexity: taking advantage of context and relationships in dissemination of school-based interventions. Health Promot Pract.

[33] Lavis JN, Lomas J, Hamid M, Sewankambo NK (2006). Assessing country-level efforts to link research to action. Bull World Health Organ.

[34] LAVIS JOHN N., ROBERTSON DAVE, WOODSIDE JENNIFER M., McLEOD CHRISTOPHER B., ABELSON JULIA (2003). How Can Research Organizations More Effectively Transfer Research Knowledge to Decision Makers?. Milbank Quarterly.

[35] Ward V, House A, Hamer S (2009). Developing a framework for transferring knowledge into action: a thematic analysis of the literature. J Health Serv Res Policy.

[36] Kitson AL (2009). The need for systems change: reflections on knowledge translation and organizational change. J Adv Nurs.

[37] Davison CM (2009). Knowledge translation: implications for evaluation. New Dir Eval.

[38] Court J, Young J (2006). Bridging research and policy in international development: an analytical and practical framework. Dev Pract.

[39] Stockton L, Riley B, Willis C, Zummach D. Scaling up cancer and chronic disease prevention interventions for population health impact: report on a planning meeting. Waterloo; 2015. https://uwaterloo.ca/propel/sites/ca.propel/files/uploads/files/scaling-up_meetingreport_accessible_noappendices.pdf. Accessed 13 Sept 2018.

[40] Pearson M, Brand SL, Quinn C, Shaw J, Maguire M, Michie S (2015). Using realist review to inform intervention development: methodological illustration and conceptual platform for collaborative care in offender mental health. Implement Sci.

[41] Kastner M, Perrier L, Hamid J, Tricco AC, Cardoso R, Ivers NM (2015). Effectiveness of knowledge translation tools addressing multiple high-burden chronic diseases affecting older adults: protocol for a systematic review alongside a realist review. BMJ Open.

[42] Saul JE, Willis CD, Bitz J, Best A (2013). A time-responsive tool for informing policy making: rapid realist review. Implement Sci.

[43] Carrey NJ, Curran JA, Greene R, Nolan A, McLuckie A (2014). Embedding mental health interventions in early childhood education systems for at-risk preschoolers: an evidence to policy realist review. Syst Rev..

[44] Tchameni Ngamo S, Souffez K, Lord C, Dagenais C (2016). Do knowledge translation (KT) plans help to structure KT practices?. Health Res Policy Syst..

[45] Brett J, Staniszewska S, Mockford C, Herron-Marx S, Hughes J, Tysall C (2014). Mapping the impact of patient and public involvement on health and social care research: a systematic review. Health Expect.

[46] Rycroft-Malone J, Burton CR, Bucknall T, Graham ID, Hutchinson AM, Stacey D (2016). Collaboration and co-production of knowledge in healthcare: opportunities and challenges. Int J Health Policy Manag.

[47] Heaton J, Day J, Britten N (2016). Collaborative research and the co-production of knowledge for practice: an illustrative case study. Implement Sci.

[48] Voorberg WH, Bekkers VJJM, Tummers LG (2015). A Systematic review of co-creation and co-production: embarking on the social innovation journey. Public Manag Rev.

[49] Bowen SJ, Graham ID (2013). From knowledge translation to engaged scholarship: promoting research relevance and utilization. Arch Phys Med Rehabil.

[50] Armstrong R, Waters E, Crockett B, Keleher H (2007). The nature of evidence resources and knowledge translation for health promotion practitioners. Health Promot Int.

