# Supplementary material for: Knowledge translation for realist reviews: a participatory approach for a review on scaling up complex interventions
Source: Health Res Policy Syst. 2018 Oct 22;16:101. doi: 10.1186/s12961-018-0374-1 (PMC6198505; doi:10.1186/s12961-018-0374-1)
Supplement: Supplementary file 1 — International meeting agenda. Questions from consultations with knowledge users. Summary of papers considered relevant to understanding the knowledge translation (KT) strategy for realist reviews. (DOCX 30 kb) [file 12961_2018_374_MOESM1_ESM.docx]

**Supplementary Material**

1. International meeting agenda

**Meeting Agenda**

**Scaling up cancer and chronic disease prevention interventions for population**

**Meeting objectives:**

1. Compare and contrast preliminary findings from a CIHR-funded knowledge synthesis on scaling up complex interventions with knowledge user experiences,
2. Refine knowledge translation plans for study findings, and
3. Identify some scaling up initiatives that may be promising to study as ‘natural experiments’

*** Dates, times, locations and names have been omitted.*

| Welcome | Welcome to meeting  Introduction of team members and participants  Purpose and structure of meeting |
| --- | --- |
| Insights from practical experiences | Two user panel members will present and discuss their scaling up experiences, including reflections on:   - What was scaled up? - What did ‘scaled up’ mean in this example? Where did the initiative start and where is it now? - What actions supported scaling up? - What enabled or constrained those actions (e.g. contextual factors)?   Other participants will have the opportunity to share and discuss their own scaling up experiences |
| Insights from scaling up literature | Overview of realist review methods  Presentation of preliminary findings from the CIHR knowledge synthesis |
| Compare and contrast practical insights with literature | Discussion to identify themes to explore in more detail in session 3:   - What mechanisms are apparent in both the practical insights and the literature? What seem to be disconnects between mechanisms firing in the practical insights and the literature? - What themes related to scaling up actions are apparent in both the practical insights and the literature? What seem to be disconnects between actions in practice and the literature? - What other themes related to scaling up stand out to you as common or divergent between practice and the literature? |
| An emerging action learning agenda on scaling up | Table talks to discuss and explore key themes related to scaling up (identified during the morning sessions):   - What do we need to learn about this theme? - What are examples of scaling up that could provide some insights on this theme? |
| An emerging action learning agenda on scaling up | Large groups reports and feedback from table talks |
| Refining KT plans | Overview of KT plans proposed in CIHR synthesis grant  Questions for discussion:   - How might you use the results of the review? - Who else might use the results? - What results do you think will be most useful? - How might the KT plans be refined? |
| Summary of meeting, next steps and thanks | Closing remarks  Round table reflections from all participants:   - What was one new insight from this meeting? - What is one action you will take as a result of the meeting? |

1. Questions from consultations with knowledge users
2. From your perspective, who are the primary audiences, either individuals or groups, in your organization that you think could benefit from the scaling up results?
   1. For each audience you identified, for what purpose would you expect them to use the results?
3. Which results from the review would be most pertinent to [each person or group]?
4. Are there individual characteristics [of each person/group] that we should be aware of that may influence the use or uptake of the results?
5. Are there existing organizational conditions / context we should be aware of?
6. When thinking about presenting findings to [individual/group] – what would be some considerations to keep in mind?
7. Are there other factors that would influence the use of the results for [individual/group]?
8. What is one KT product or event on the results of the review that you would like for your organization?
9. Realist methodology seeks to answer under what conditions specific mechanisms for change are activated and lead to certain outcomes.

Our research question was: What are patterns and pathways for scaling up population interventions, with a particular focus on relationships between scaling up actions and contextual factors, mechanisms by which actions are effective, and outcomes?

1. At the outset of this project, how did you envision the results from the synthesis?
2. From a practical perspective, how could the results from a realist synthesis be described and communicated in order to be most useful to your organization?
3. Summary of papers considered relevant to understanding KT strategy for realist reviews

| **Article** | **Strategies to translate findings into action** | **Intended use of findings** | **Stakeholder involvement** | **Challenges** | **Enablers** |
| --- | --- | --- | --- | --- | --- |
| (32) | A conceptual platform that specifies the core processes of how an integrated, person-centred system to improve the mental health of offenders with common mental health problems is proposed to work. | Inform the development of an intervention to improve collaborative mental health care for offenders with common mental health problems  Help stakeholders think differently about how mental health care for offenders is organized, designed and delivered. | Engagement of people with lived experiences, practitioners, policymakers and researchers in the synthesis process. | Conceptual platform does not include resource implications of changes in service delivery. | Engagement of a variety of stakeholders. |
| (33) | Share with a wide range of knowledge users and stakeholders through publications, presenting work at geriatric and disease-specific conference, lay publications, and workshops with key stakeholders.  Develop a tool to facilitate ongoing, proactive and preventative support for optimal disease management for seniors with multiple chronic diseases. | Improve the care of seniors with multiple chronic diseases | Consultations with experts among the team in developing the programme theory. | N/A | N/A |
| (34) | Present to policymakers with a focus on groupings of interventions that can be implemented within their context and can be easily acted on.  Present program theories as a way to understand: how changes in context may interact with mechanisms to produce outcomes of interest; and, potential unintended consequences resulting from changes in context and their resultant interactions with mechanisms. | Use of rapid realist reviews to produce a product that is useful to policy makers in responding to time-sensitive and/or emerging issues, while preserving the core elements of realist methodology. | Local reference group of knowledge users, as well as an expert panel involved throughout the review process. | Rapid feedback from both reference group and expert panel members. | Reference group to engage key agency or government staff in the process without requiring excessive time commitments.  Honoraria for expert panellists.  Opportunities to be involved in dissemination of the work through conference presentations, papers, etc.  Highlight intervention and outcomes links in reports, and use theory to explain why those interventions might work well in a given context for specific populations. |
| (35) | Knowledge users and researchers worked together to identify stakeholder groups and develop recommendations taking into account the context and implementation chains of the different stakeholder groups.  An online brainstorming exercise to identify potential barriers for the uptake of the recommendations. A dissemination plan addressed the barriers and included strategies that have worked with the stakeholder groups in the past.  Knowledge broker who supports dissemination process including merging policy and academic perspectives, as well as crafting recommendations that are sensitive to local policy context. | Evidence-based mental health programming, policy and interventions for preschoolers at high risk for socio-emotional challenges. | Knowledge users and researchers worked together through each stage of the review process. | N/A | N/A |
